# Predictors of suicide ideation among older adults with bipolar disorder

**DOI:** 10.1371/journal.pone.0187632

**Published:** 2017-11-16

**Authors:** Norm O’Rourke, Marnin J. Heisel, Sarah L. Canham, Andrew Sixsmith, BADAS Study Team

**Affiliations:** 1 Department of Public Health and Center for Multidisciplinary Research in Aging, Ben-Gurion University of the Negev, Be’er Sheva, Israel; 2 Department of Psychiatry University of Western Ontario and Lawson Health Research Institute, London, Ontario, Canada; 3 Gerontology Research Centre, Simon Fraser University, Vancouver, British Columbia, Canada; 4 STAR Institute, Simon Fraser University, Surrey, British Columbia, Canada; 5 IRMACS Centre, Simon Fraser University, Burnaby, British Columbia, Canada; Chiba Daigaku, JAPAN

## Abstract

**Objectives:**

Bipolar disorder (BD) carries the greatest risk of death by suicide of all psychiatric conditions as 25%–50% of those with BD will make one or more suicide attempt, and about 15% will intentionally end their lives. Among young adults with BD, substance misuse, medication non-adherence, age at onset, and comorbid psychiatric conditions each predict self-harm. It is currently unclear if these same factors or others predict suicide ideation among older adults with BD.

**Methods:**

We recruited a global sample of 220 older adults with BD over 19 days using socio-demographically targeted, social media advertising and online data collection (*Mean* = 58.50, *SD* = 5.42; range 50 to 81 years). Path analyses allowed us to identify direct and indirect predictors of suicide ideation among older adults with BD.

**Results:**

Cognitive failures (perception, memory, and motor function), depressive symptoms, alcohol misuse, and dissatisfaction with life as direct predictors of suicide ideation; duration of BD symptoms and medication non-adherence emerged as indirect predictors. Of note, the significant impact of sleep on suicide ideation is indirect via depressive symptoms, cognitive failures, medication non-adherence and life dissatisfaction.

**Conclusions:**

As with young adults with BD, alcohol misuse and medication non-adherence emerged as significant predictors of suicide ideation. In addition, cognitive failures directly and indirectly predict suicide ideation in this sample of older adults with BD. Population aging and treatment efficacy are leading to ever growing numbers of older adults with BD. Both direct and indirect predictors of suicide ideation need to be considered in future BD research and treatment planning.

## Introduction

Rates of suicide by those with bipolar disorder (BD) are roughly 20 times greater than the general population [1]. In fact, BD carries the greatest risk of death by suicide than all other psychiatric conditions [2]. Among younger adults with BD, earlier age of onset, psychiatric comorbidity, alcohol misuse [3], and a recent depressive episode [4] are each significant predictors of death by suicide. In contrast, mania appears largely unrelated to intentional self-harm [5].

Over recent decades, there has been a large increase in numbers of older adults with BD [6]. This is likely due to overall population aging as well as the efficacy of pharmacotherapy compared to generations past. To date, however, it is unclear if the same factors that predict self-harm by young adults with BD are the same for their older counterparts [7–8]. For instance, cognitive loss and sleep disturbance are known suicide risk factors with older adults generally [9–11]; both of these are prevalent in later life with BD [7,12].

The recent meta-analysis by the International Society for Bipolar Disorders Task Force (ISBDTF) on Suicide [2] concluded that younger age at onset, illicit substance use (not marijuana), and depressive symptoms (current and first illness episode) significantly predict suicide attempts by those with BD. Of note, this meta-analysis did not consider age as either a protective or risk factor. Yet there may be neurocognitive factors germane to later life with BD (e.g., impaired decision making) that further increase suicide risk [2]. For this study, we examined predictors of suicide ideation specifically with older adults with BD. We define ‘later life’ BD as 50+ years of age as recommended by the ISBDTF [8].

## Methods

### Participant recruitment

As part of the BADAS (Bipolar Affective Disorder and older Adults) Study, we recruited a sample of 220 older adults with BD mostly from Canada, the U.S., U.K., Ireland, Australia, and New Zealand [13]. This was achieved over 19 days using social media advertising micro-targeting those with BD [14]. Participants were drawn from a global population of approximately 6.2 million English-speaking, adult Facebook users with ‘bipolar disorder interests’ (e.g., members of online BD support networks). The BADAS study is being undertaken with ethics approval from Simon Fraser University, Burnaby (BC), Canada.

By clicking on advertisements appearing along the sidebar within Facebook or embedded within newsfeeds, prospective participants were directed to an online consent form that specified study inclusion criteria. Thereafter they completed a series of counterbalanced online questionnaires hosted on a secure university https server; responses were encrypted before transmission [13].

This methodology avoids Berkson’s bias in which samples composed of more severely impaired or refractory patients from ambulatory clinics do not reflective the population of persons with BD [3]. For instance, in our sample there is a subset who avoid clinical contact. Many have discontinued pharmacotherapy and instead self-medicate with alcohol and marijuana [15]. These social media algorithms are highly effective at micro targeting prospective study participants and maybe as importantly, effectively exclude those who do not meet study inclusion criteria [14].

### Participant descriptive features

This sample was composed of 140 women and 80 men, ranging in age from 50 to 81 years (Mean = 58.50, *SD* = 5.42). The largest proportion lived in North America (36% from Canada, 32% from the U.S.), while 16% were from the U.K., 6% from Australia, 5% from Ireland, and 2% from New Zealand. In total, participants from 10 countries provided responses. Participants required 32 minutes on average to complete online questionnaires (*SD* = 30.79).

Of those reporting a specific diagnosis, 40% indicated that they had been diagnosed with BD II, 32% had been diagnosed with BD NOS (not otherwise specified), and 20% reported a BD I diagnosis. Most participants were currently taking one or more mood stabilizer (58.9%) and antidepressant (64.4%); smaller numbers were currently prescribed an anxiolytic (38.9%) or antipsychotic medication (38.3%). And most indicated that they had made one or more suicide attempt in the past (53.2%), which is higher than reported with younger adults samples [16]. We speculate that this is due to the age of participants (i.e., greater number of years with BD lived experience).

### BD diagnosis—Confirmation analyses

To corroborate that participants were in fact older adults with BD, they were asked to list any prescribed psychotropic medications by category as well as comorbid psychiatric conditions. Of those specifying medications, 95% correctly listed and categorized both mood stabilizers and antidepressants, and 84% for antipsychotics. Also, comorbid conditions and the relative frequency reported by participants correspond to epidemiological BD research (i.e., anxiety disorders were most commonly cited) [17]. Given participants’ ability to specify their prescribed medications with such accuracy, we contend it is unlikely that participants invented or mispecified their BD diagnosis. In no small degree, this is due to the fact that social media advertisements were micro-targeted specifically to older adults with BD and not other populations.

### Instruments

#### Geriatric suicide ideation scale (GSIS)

We assessed the presence and severity of suicide ideation using the Geriatric Suicide Ideation Scale (GSIS) [18], a 31-item multidimensional measure which has been developed and validated explicitly among older adults. GSIS items are scored on a Likert scale, ranging from 1 (strongly disagree) to 5 (strongly agree) [19–21]. The GSIS also contains an item inquiring whether the respondent has attempted suicide in the past, a primary risk indicator for self-harm in future [22].

GSIS responses demonstrate strong internal consistency (.90 < α < .97), test-retest reliability over short (e.g., 2–4 weeks) and longer (e.g., 1–2 years) periods of time [18, 23–25], Concurrent and discriminant validity has been demonstrated vis-à-vis depression, hopelessness, impulsivity, and (the absence of) psychological well-being and life dissatisfaction with clinical, community-residing older adults [26–28]. The GSIS has been validated for use with older adults with BD [29].

#### Patient health questionnaire (PHQ-9)

The PHQ-9 [30] is a self-report version of the depression module from the PRIME-MD diagnostic interview [31]. Participants respond to PHQ-9 items (4 response options) based on problems they have experienced over the past two weeks. A total score greater than 9 suggests significant symptomology with 89% sensitivity and 88% specificity vis-à-vis diagnosis of a major depressive episode [32]. The PHQ-9 has been used widely with unipolar and bipolar patient populations [33].

#### Cognitive failures questionnaire (CFQ)

Persons with BD commonly report and exhibit more cognitive difficulties than others [34]. This is especially true for older adults with BD who appear to exhibit greater age-associated cognitive decline [35]. It has been suggested that increased cortisol activity during major depressive episodes may drive cumulative excitotoxicity in the amygdala and other brain regions associated with cognitive loss [36]. This however remains a point of contention [37–38]. In their recent review, Sajatovic and colleagues [8] contend that cognition is a possible confound that needs to be considered in BD research with older adults.

For this study, participants completed the Cognitive Failures Questionnaire (CFQ) [39] to allow us to identify associations between cognitive failures and suicide ideation. The CFQ is a self-report measure of perceived failures in perception, memory, and motor function. CFQ responses correlate positively with accident proneness, human error and psychological strain, and inversely with executive functioning [40]. Test-retest reliability over 2-years has been reported as *r* = .71 [41].

CFQ responses are inversely associated with quality of life, even after controlling for depressive symptoms and objective indices of cognitive loss [42]. Among older adults, CFQ responses may be an early indicator of neurodegeneration, detectable prior to objective indices of cognitive decline [43].

#### Medication adherence scale (MAS)

Medication non-adherence is associated with reduced psychosocial functioning and BD disease severity [44]. With this in mind, participants completed the 8-item MAS [45]. Factors associated with MAS responses include depressive symptoms, socioeconomic status, family support, alcohol consumption [46], and belief in the efficacy of treatment [47].

#### Pittsburgh sleep quality index (PSQI)

Poor sleep is a common feature of BD associated with both hypo/manic and depressive episodes, and quality of life [48]. The 19-item PSQI [49] is a self-rated questionnaire that assesses perceived sleep quality over the past 30 days. As reported by Saunders and colleagues [50], each of these domains differs significantly between euthymic persons with BD and control participants. PSQI responses are also significantly correlated with cognitive loss with BD [51].

#### Alcohol use disorders identification test (AUDIT)

The AUDIT [52] is a 10-item self-report scale initially developed to measure alcohol misuse in primary healthcare. Internal consistency of responses ranges from .74 < α < .96. Concurrent validity has been demonstrated in comparison to the Michigan Alcoholism Screening Test (*r* = .77) [53].

Research with the AUDIT demonstrates that cognitive loss is greater for bipolar patients with concomitant alcohol use disorders [54]. The AUDIT has also been used widely with older adults [55] although various authors contend that the problem drinking threshold is lower for older than young adults due, in part, to age-related changes in alcohol metabolism [56].

#### Satisfaction with life scale (SLS)

The SLS [57] measures perceived quality of life on the basis of person-specific criteria. Respondents compare their current circumstances against subjective standards to arrive at a global appraisal of life satisfaction [58]. Participants respond to five questions with seven response alternatives ranging from 1 (very strongly disagree) to 7 (very strongly agree). Good internal consistency has been reported with older adults (α = .82) [23, 59]. Previous research indicates that depression is associated with lower life satisfaction whereas the SLS is unrelated to hypo/manic symptoms of BD [60].

### Hypotheses and analytic strategy

We performed a series of path analyses to identify direct and indirect predictors of suicide ideation. Consistent with prior BD research, we hypothesized that depressive symptoms, alcohol misuse, duration of BD diagnosis, number of comorbid psychiatric diagnoses, and lower levels of life satisfaction would predict suicide ideation [1–5]. In accord with research with general older adult populations, we assumed that cognitive failures and sleep quality would also emerge as significant predictors of suicide ideation [61].

Path analysis is an extension of linear regression with three significant advantages. 1) Path analysis allows us to simultaneously predict one or more dependent variables (touched by arrowhead in path models). Arrows pointing from independent to dependent variables represent significant prediction (i.e., critical ratio values > |1.96|). Path analysis is a multivariate statistical procedure meaning that all significant paths emerged concurrently (i.e., over and above other statistically significant results).

2) Path models allow us to identify both direct and indirect predictors of suicide ideation. Indirect prediction occurs via other variables (i.e., 2+ pathways between variables). In complex or more nuanced path models, variables can have direct and indirect effects on dependent variables, and indirect effects can be of equal or greater magnitude than direct effects (total effects = direct + indirect effects).

3) Computing path analyses with structural equation modeling (SEM) software allowed us to obtain goodness of fit information for the overall model. Good model fit is required to interpret individual results. We report three goodness-of-fit-indices to assess the overall fit of path models: An incremental, an absolute, and a parsimonious fit index.

The Comparative Fit Index (CFI) is an *incremental index* representing the extent to which a hypothesized model is a better fit to data than the null model. Coefficient values greater than .94 for the CFI indicate good model fit [62]. The Standardized Root Mean Square Residual (SRMR) is an *absolute index* which represents the standardized difference between observed and predicted correlations within a hypothesized model. Finally, the Root Mean Square Error of Approximation (RMSEA) is a *parsimony index* which represents the extent to which a hypothesized model fits data relative to the general population. Coefficient values less than 0.055 for the SRMR and RMSEA, indicate good model fit [63].

Path analyses were performed for this study in a 3-step process. A baseline model was first computed in which all independent variables were assumed to directly predict suicide ideation; all nonsignificant paths were deleted. Lastly, unhypothesized but statistically significant paths are added to the model where justified based on theory or prior research [63].

## Results

Older men and women recruited for this study were demographically and clinically similar. They did not differ in age, years of education, duration of BD diagnosis, number of comorbid psychiatric diagnoses, or number of prescribed psychotropic medications. Nor did they differ in number of prior suicide attempts, χ^2^(*df* = 1) = 1.52, *p* = .28. These descriptive statistics suggest considerable symptom similarity between older men and women with BD. (Table 1)

**Table 1:** Descriptive statistics and study variables.

	Men (*SD*)	Women (*SD*)	range	*t* value	Alpha (α)
Age	58.69 (5.51)	57.75 (5.19)	50–81	.91	
Education	12.00 (6.56)	12.45 (5.42)	1–28	.40	
Duration D_x_	13.75 (11.30)	14.32 (10.38)	.12–46	.26	
Comorbid D_x_	.81 (1.23)	1.11 (1.26)	0–5	1.24	
Total Ψ R_x_	2.31 (1.27)	2.53 (1.41)	0–7	.80	
Suicide Ideation	75.54 (28.02)	76.55 (26.03)	38–147	.27	.85
Depressive S_x_	16.24 (8.94)	18.23 (7.72)	0–31	1.26	.92
Alcohol Misuse	4.88 (5.17)	4.07 (5.43)	1–32	.78	.86
R_x_ Adherence	4.26 (2.10)	4.86 (3.01)	0–12	1.15	.74
Cognitive Failures	51.33 (18.60)	55.88 (19.03)	3–100	1.25	.94
Sleep Quality	8.93 (2.81)	9.40 (3.11)	3–17	.88	.70
Life Satisfaction	16.81 (8.77)	15.45 (7.15)	5–35	.67	.89

*Note*. Duration D_x_ = years since BD diagnosis, Comorbid D_x_ = number of comorbid psychiatric conditions, Total Ψ R_x_ = number of prescribed psychotropic medications. Suicide ideation = GSIS, Depressive S_x_ = PHQ-9, Alcohol misuse = AUDIT, R_x_ adherence = MAS, Cognitive failures = CFQ, Sleep quality = PSQI, Life satisfaction = SWL.

Overall levels of suicide ideation are high in this sample of older adults with BD, Mean = 78.18, SD = 22.89. By comparison, the average GSIS score was 67.40 (SD = 13.82) in psychiatric inpatient and outpatients [19]; and among suicidal older adults and those who have made a recent attempt, Mean = 79.52 (SD = 24.22). The mean for this BD sample is approximately ½ SD above a general sample of older mental health patients, and equivalent to older outpatients currently receiving treatment for suicidal ideation and behavior [25]. As previously noted, 53.2% of these participants reported having made 1+ suicide attempt.

Reported levels of suicide ideation did not significantly differ between men (Mean = 75.54, *SD* = 28.02; α = .84) and women Mean = 76.55, *SD* = 26.03; α = .85, *t*(*df* = 215) = .79, *p* = .79. Nor did depressive symptoms differ by sex. Similarly, medication adherence, alcohol misuse, cognitive failures, sleep quality, and life satisfaction were similar for men and women. We found no between-sex differences in socio-demographic or BD related factors, including suicide ideation.

### Predictors of suicide ideation

We computed path analyses to identify predictors of suicide ideation in this sample of older adults with BD. As with younger adults, we hypothesized that depressive symptoms, alcohol misuse, duration of BD diagnosis, number of comorbid psychiatric diagnoses, and life dissatisfaction would predict GSIS responses. We further assumed that cognitive failures and poor sleep would predict suicide ideation as found with other older adults [10]. Yet a more nuanced model emerged with both direct and indirect predictors of suicide ideation, and some predictors having both direct and indirect effects (i.e., via other variables). With 220 participants and 7 independent variables, statistical power exceeded .80 enabling us to identify medium to large effect sizes (α < .01) [63].

**Fig 1:**
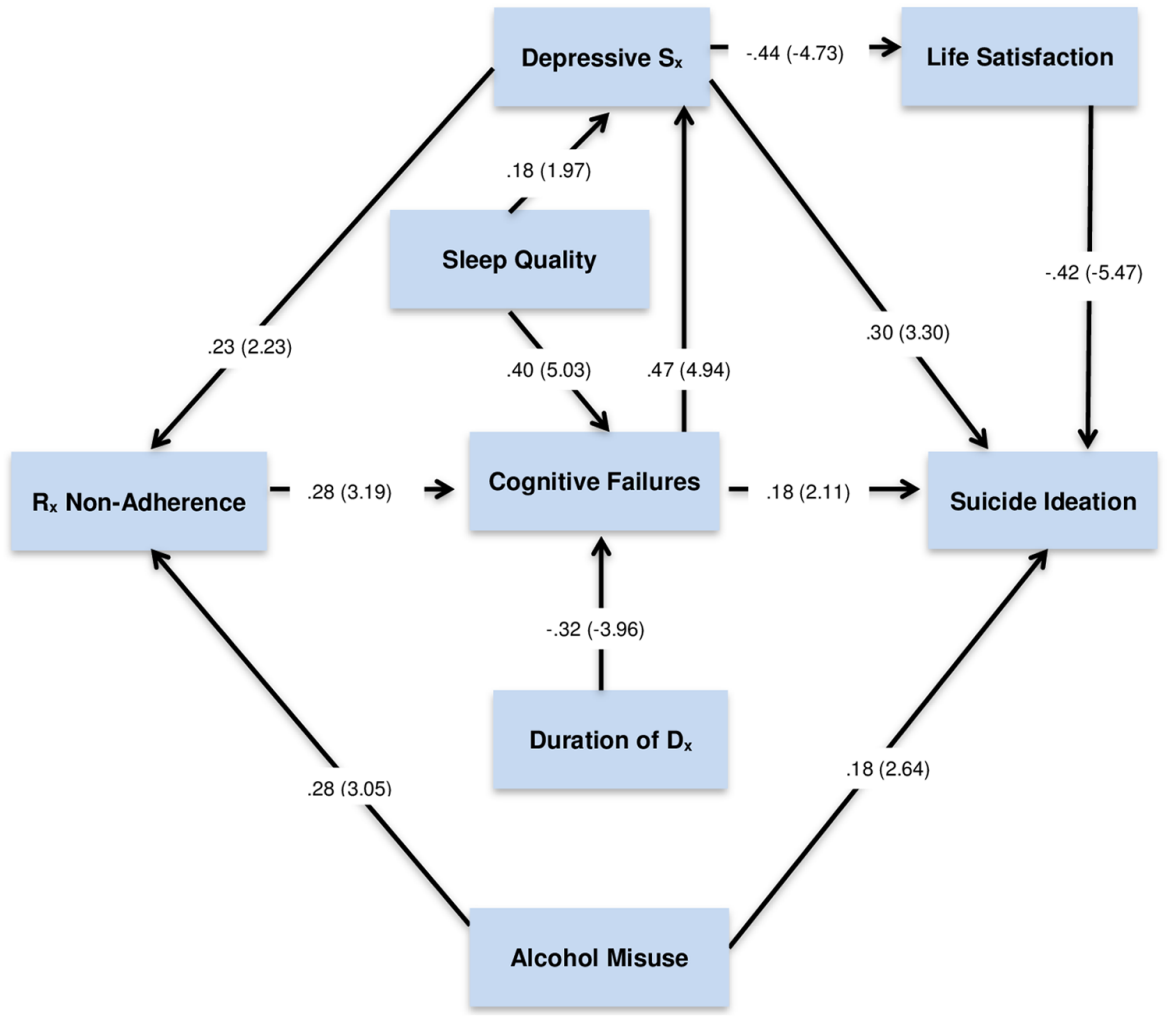
Path analytic model of predictors of suicide ideation among older adults with bipolar disorder. *Note*. Suicide ideation = GSIS, Depressive S_x_ = PHQ-9, Alcohol misuse = AUDIT, R_x_ adherence = MAS, Cognitive failures = CFQ, Sleep quality = PSQI, Life satisfaction = SWL. Parameters expressed as maximum likelihood estimates (standardized solution). Parenthetical numbers indicate significance levels for parameter estimates (statistically significant critical ratios values > |1.96|).

Goodness of fit indices were within optimal parameters for the resultant model, χ^2^(*df* = 16) = 13.39, *p* = .84. The Comparative Fit Index (CFI ≥ .95; CFI = .99), the Standardized Root Mean Square Residual (SRMR ≤ .055; SRMR = .044), and the Root Mean Square Error of Approximation (RMSEA ≤ .05; RMSEA = .001) were each in ideal parameters. The full 90% confidence interval for the RMSEA was within acceptable parameters (0 < RMSEA CL_90_ < .080). (Fig 1)

Depressive symptoms, alcohol misuse, cognitive failures, and lower life satisfaction directly predicted suicide ideation. Depressive symptoms (via life satisfaction) and cognitive failures (via depressive symptoms) also emerged as indirect predictors. Medication non-adherence, and duration of BD diagnosis also indirectly predicted GSIS responses. As found with other older adults, sleep disturbance significantly predicts suicide ideation, but indirectly via depressive symptoms, cognitive failures, and medication non-adherence. (Table 2)

**Table 2:** Direct and indirect predictors of suicide ideation among older adults with bipolar disorder (standardized coefficients).

	Duration D_x_	Sleep	Alcohol	Depress S_x_	R_x_ Adhere	Cog Fail	Life Sat
Depressive S_x_ ∘ direct effects ∘ indirect effects	-- -.15	.18 .20	-- .04		-- .13	.47 .01	
totals	-.15	.38	.04		.13	.48	
R_x_ Adherence ∘ direct effects ∘ indirect effects	-- -.04	-- .09	.28 .01	.23 .01		-- .11	
totals	-.04	.09	.29	.24		.11	
Cognitive Failures ∘ direct effects ∘ indirect effects	-.32 -.01	.40 .03	-- .08	-- .06	.28 .01		
totals	-.33	.43	.08	.06	.29		
Life Satisfaction ∘ direct effects ∘ indirect effects	-- .07	-- -.17	-- -.02	-.44 -.01	-- -.06	-- -.21	
totals	.07	-.17	-.02	-.45	-.06	-.21	
Suicide Ideation ∘ direct effects ∘ indirect effects	-- -.13	-- .26	.18 .04	.30 .21	-- .12	.18 .24	-.42 --
totals	-.13	.26	.22	.51	.12	.42	-.42

*Note*. Suicide ideation = GSIS, Depressive S_x_ = PHQ-9, Alcohol misuse = AUDIT, R_x_ adherence = MAS, Cognitive failures = CFQ, Sleep quality = PSQI, Life satisfaction = SWL. Parameters expressed as maximum likelihood estimates.

The absence of life satisfaction was the strongest direct predictor of suicide ideation in this sample. However, the combined direct and indirect effects of cognitive failures were of equal magnitude to dissatisfaction with life. The combined direct and indirect effects of depressive symptoms contributed most to prediction of GSIS responses. Like cognitive failures, depressive symptoms directly and indirectly contribute to prediction of suicide ideation. With cognitive failures, the indirect effect on suicide ideation (via depressive symptoms) is greater than its direct effect.

Alcohol misuse and depressive symptoms also predicted medication non-adherence, but not vice versa. In other words, depressive symptoms appear to be a cause, not a consequence of medication non-adherence in this cross-sectional sample of older adults with BD. This suggests that participants may self-medicate with alcohol in place of prescribed medications. Moreover, sleep quality appears to mediate the association between cognitive failures and depressive symptoms. Although poor sleep does not appear to be a direct predictor of suicide ideation, the indirect effect is substantive (e.g., greater than the combined effects of alcohol misuse). Moreover, the contribution of poor sleep is greater than that of medication non-adherence or duration of BD diagnosis. Alcohol misuse is the sole direct predictor of medication non-adherence in this sample.

Overall, findings indicate that both BD and later life factors contribute to prediction of suicide ideation in this older adult sample. Associations are not always direct; and except for alcohol misuse, predictors are significantly mediated by other factors. For instance, both poor sleep and cognitive failures augment depressive symptomatology, which, in turn, further increases suicide ideation. Fully 51% of observed variance in GSIS responses is explained in this path model meaning that direct and indirect predictors account for more than half of all variance in suicide ideation (*R*^2^ = .51, *p* < .01).

## Discussion

This study examined predictors of suicide ideation among adults aged 50+ years with BD. One finding of note is the considerable similarity of socio-demographic and symptomatology between older men and women with BD recruited for this study. The majority of both groups reported that they had made one or more suicide attempt, and both reported similar levels of suicide ideation (and depressive symptoms). This is in contrast to unipolar depression in which women report greater symptomatology and are more likely to self-harm, while men are more likely to die by suicide [64–65]. This finding is in accord with clinical research and practice indicating that symptom patterns are generally similar for men and women with BD [66]. This appears to extend to later life with BD.

A further finding of note is the inverse association between duration of diagnosis and both depressive symptoms and suicide ideation. With younger adults, earlier age of BD onset has been identified as a suicide risk factor [2]. This may suggest that older adults with BD have acclimated to their diagnosis and symptomatology, and have devised (effective) coping strategies over time.

Yet it is noteworthy that this appears to be a highly dysphoric sample: the majority of both men and women reported one or more suicide attempt, depressive symptomatology was high, and life satisfaction was low. Older adults with BD may be a distinct subset of persons with this disorder who survive to later life.

Results of this study replicate and extend findings reported with younger adults with BD. Factors such as alcohol misuse, medication non-adherence, and depressive episodes and symptoms each predict suicide ideation and self-harm. In addition, later life factors such as sleep quality and cognitive failures also emerged as significant predictors of suicide ideation, both directly and indirectly. Future research should examine the role of substances other than alcohol in suicide ideation, as illicit substance use is common with BD [67]. This is germane as the current cohort of baby boomers have had greater exposure to and use of illicit substances than generations past [68].

More research is also required to clarify the extent to which perceived and objective cognitive loss contributes to suicide ideation and behavior by older adults with BD. Our findings also suggest that cognitive failures are a significant direct and indirect predictor of suicide ideation (i.e., impact upon depressive symptoms). Studies should be undertaken to identify which aspects of cognition are associated with suicide ideation (e.g., memory loss vs. executive functioning) and the potential role of resiliency in preventing suicide in later life [20]. Further research is required to identify ways in which suicide ideation among older adults is similar to and different from younger adults and why.

### Study limitations and directions for future study

We were able to recruit an international sample of older adults with BD for this study in 19 days. And though we avoided the biases inherent in recruitment via psychiatric clinics [3], online data collection remains a novel research methodology [14]. Future research should set out to replicate this model with participants recruited via this and more traditional means.

And though we recruited a sample of older adults with BD, at 58.5 years of age on average (*SD* = 5.42) this is a young, older adult sample. Yet it is worth noting that we were able to recruit participants over 80 years of age using social media. Use of these technologies will only increase as baby-boomers with and without mental illness enter late life.

Future research should also undertake longitudinal data collection and clinical assessment of study participants (e.g., confirmation of BD diagnoses). In this cross-sectional model, depressive symptoms predict reduced medication adherence in the moment. Yet longitudinal analyses are more likely to reveal the reverse association (i.e., non-adherence predicts mood episode relapse over time).

The results of this study have implications for clinical research and practice. Clinicians are advised to directly assess the presence and severity of suicide ideation among older adults with BD to determine the contribution of dysphoria, substance misuse, cognition, and medication use to suicide risk. And though its association appears indirect, the impact of sleep disturbance on suicide ideation is considerable (i.e., via depressive symptoms, cognitive failures, medication non-adherence). Clinical intervention research is required examining older adults at-risk [69], including those with BD and other mental health conditions that significantly increase the risk of self-harm.
